# Twig and Leaf Morphological Traits and Photosynthetic Physiological Characteristics of *Periploca sepium* in Response to Different Light Environments in Taohe Riparian Forests

**DOI:** 10.3390/plants15020179

**Published:** 2026-01-07

**Authors:** Min Ma, Chengzhang Zhao, Qun Li, Gang Hou, Junxian Chen

**Affiliations:** 1 College of Life Science, Northwest Normal University, Lanzhou 730070, China; mamin5252008@163.com; 2 College of Forestry, Gansu Agricultural University, Lanzhou 730070, China; 3 College of Geography and Environmental Science, Northwest Normal University, Lanzhou 730070, China; 4 College of Geography and Environmental Science, Northwest Normal University, Research Center of Wetland Resources Protection and Industrial Development Engineering of Gansu Province, Lanzhou 730070, China; 5 Gansu Lintao Taohe National Wetland Park Administration, Lintao 730500, China

**Keywords:** twig and leaf morphological traits, photosynthetic physiological characteristics, environmental response, *Periploca sepium*, riparian forests

## Abstract

Understanding the variations in twig and leaf morphologies and photosynthetic physiological characteristics of riparian forest plants in heterogeneous habitats is of great significance for revealing their phenotypic plasticity mechanisms and ecological adaptation strategies. In this study, the riparian forest plant *Periploca sepium * Bunge was selected as the research object. According to the canopy light environment experienced by the *P. sepium* population, three habitats were established: under-canopy, gap, and full-sun areas. We studied the twig and leaf morphological and photosynthetic characteristics of *P. sepium* under heterogeneous light environments, as well as the relationships between these two aspects. Plants in the under-canopy area developed long and thick twigs with few large leaves, coupled with high actual photosynthetic efficiency of photosystem II (Y(II)) and low non-photochemical quenching (NPQ), whereas those in the full-sun area exhibited the opposite covariation strategy. Significant correlations between twig and leaf morphologies and photosynthetic physiological characteristics were found across all habitats. The coordinated variations in twig and leaf morphologies and photosynthetic physiology of *P. sepium* embody a resource investment trade-off strategy that plants have evolved through long-term adaptation to heterogeneous light environments.

## 1. Introduction

Light is a highly variable environmental factor in natural forests, and it profoundly affects plant survival, growth, and reproduction [1,2]. Due to the complex architecture of tree canopies, light intensity in forest habitats results in considerable small-scale spatial heterogeneity [3,4]. Canopy shading leads to uneven spatial distribution of light within the community, alters the photosynthetically active radiation received by the canopy, and creates differentiated community environments. In the face of such heterogeneous habitats, plants adopt local rapid adaptation to avoid environmental pressures, eventually forming adaptive strategies in both external morphology and internal physiology [5].

Plant functional traits reflect the phenotypic plasticity of plants in response to their living environment under heterogeneous conditions [6,7]. As the parts of plants that are in closest contact with the external environment, twigs and leaves express traits that holistically reflect plant adaptive and survival strategies under environmental heterogeneity [8,9]. For plants, leaves serve as the principal organs for photosynthesis, transpiration, and gas exchange; their size and number thereby exert a direct influence on plant light interception efficiency and carbon acquisition capacity [10,11]. Twigs are important channels through which plants transport water and nutrients, as well as provide mechanical support for the leaves [12,13]. In addition, twigs can regulate the spatial expansion of canopy branches and leaves by changing their length, diameter, and distribution patterns, forming a rational light capture system in order to achieve the maximum carbon return [14]. The traits of plant twigs and leaves show high synergy in biomechanical and morphological structures, and this synergy jointly affects the allocation and utilization strategies of plants for resources such as light and water [15,16,17,18,19]. Therefore, the traits of twigs and leaves serve as important bases for revealing how plants can maximize the acquisition and utilization of environmental resources under environmental stress conditions and minimize the negative impacts of environmental heterogeneity on their growth.

Photosynthesis, as the central metabolic process driving plant growth and development, embodies both the adaptive strategies plants employ in response to environmental fluctuations and their dynamic interplay with the surrounding habitat [20,21]. Chlorophyll fluorescence is intricately linked to photosynthetic characteristics and serves as an integrated indicator of the plant’s photosynthetic performance [22,23]. Captured light energy is partitioned between two primary pathways: for photochemistry, which drives sugar synthesis and life-sustaining processes, and for dissipation as fluorescence or heat, a protective mechanism against photodamage [24]. Chlorophyll fluorescence parameters can reflect the changes during the photosynthetic process and are widely used as indicators of photosynthetic activity and behavior [25]. In the life cycle of plants, differences in resource niches limit the abilities of plants to obtain key growth resources, which, in turn, lead to changes in leaf morphology and photosynthetic physiological characteristics. The differences in photosynthetic physiological traits among individuals not only reflect the feedback mechanism of plant physiological traits on leaf morphology but also demonstrate the adaptability of plants to heterogeneous habitats. Analysis of photosynthetic fluorescence variations, therefore, offers critical insights into plant environmental response mechanisms and informs our understanding of the underlying survival and reproductive strategies.

As a critical ecological barrier in riparian zones, riparian forests exhibit plant community distributions, functional traits, biomass allocation patterns, and reproductive characteristics that are closely linked to regional environmental conditions [26]. *Periploca sepium*, a native plant of the Taohe riparian forest, aids in riparian biodiversity restoration and surface flood mitigation through its deep-rooted and clump-forming characteristics. The construction of Taohe Wetland Park has reduced human disturbance, and the distribution of *P. sepium* has shown a trend of expansion, spreading from full-sun areas to under-canopy and gap zones, gradually becoming the dominant shrub in these two zones; this trend of shrub encroachment is changing the structure and function of the plant community in the Taohe riparian forest by altering the competitive dynamics among plant species and affecting the light availability and resource distribution within the riparian forest. At present, studies on *P. sepium* are mainly concentrated on medicinal components [27,28], economical value [29,30,31], seed germination [32,33], adaptation to salt and drought stresses [34,35,36], and the effect of sand burial depth on seedling growth [37]; however, the response patterns of twig and leaf morphologies and photosynthetic characteristics of *P. sepium* in heterogeneous light environments remain unclear.

In view of the above considerations, *P. sepium* was investigated to examine the hypothesis that heterogeneous light environments induce adaptive changes in the plant’s twig–leaf morphologies and photosynthetic physiology. Through this study, we sought to address the following key questions: (1) What are the spatial differentiation characteristics of the twig and leaf morphological and photosynthetic physiological traits of *P. sepium* under heterogeneous habitats? (2) What are the relationships between the twig and leaf morphologies and the photosynthetic physiological characteristics of *P. sepium* in heterogeneous habitats? The aim of this study is to help reveal the phenotypic plasticity mechanisms and ecological adaptation strategies of *P. sepium* under heterogeneous habitats, and the results are expected to provide a theoretical basis for the vegetation management and protection of the Taohe riparian forest.

## 2. Results

### 2.1. Environmental Characteristics of Plant Community and Biological Characteristics in Different Light Environments

Across different light environments, the environmental characteristics of each habitat showed significant differences (*p* < 0.05, Table 1). The PAR was highest in the full-sun area and lowest in the under-canopy area, while the soil moisture content displayed the opposite trend. Moving from the under-canopy area to the full-sun area, PAR increased by 84.95%, and soil moisture content decreased by 40.15%. Due to canopy shading, environmental characteristics varied under different light conditions, thereby affecting the biological characteristics of *P. sepium*. The height and crown width of the *P. sepium* population reached their highest values in the under-canopy area and the lowest values in the full- sun area. Light conditions changed from the under-canopy area to the full-sun area, and height and crown width of the *P. sepium* population showed decreasing trends, by 23.36% and 58.27%, respectively.

**Table 1:** Environmental characteristics of plant community and biological characteristics in different light environments (mean ± SE).

Plot	Environmental Characteristics				Biological Characteristics	
	PAR	SMC	T	RH	H	CW
Under-canopy area	662.15 ± 6.52 c	23.96 ± 0.28 a	24.16 ± 0.26 b	82.98 ± 1.18 a	128.60 ± 1.71 a	1.27 ± 0.013 a
Gap area	856.55 ± 11.44 b	18.15 ± 0.13 b	24.98 ± 0.38 ab	69.04 ± 0.69 b	118.00 ± 1.75 b	0.79 ± 0.012 a
Full-sun area	1224.63 ± 10.87 a	14.34 ± 0.18 c	25.47 ± 0.37 a	50.54 ± 0.61 c	98.56 ± 1.18 c	0.53 ± 0.078 c

PAR: photosynthetically active radiation; SMC: soil moisture content; T: air temperature; RH: air relative humidity; H: height; CW: crown width. Different lowercase letters within a column indicate significant differences (*p* < 0.05) between plots (*p* < 0.05, n = 36).

### 2.2. Twig and Leaf Traits of Periploca sepium in Response to Varying Light Environments

**Figure 1:**
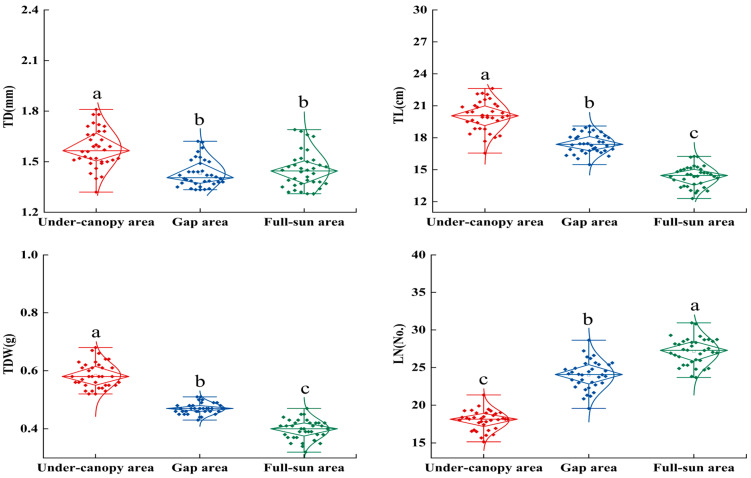
Twig traits of *Periploca sepium* in response to varying light environments. TD: twig diameter; TL: twig length; TDW: twig dry weight; LN: leaf number. Different lowercase letters denote significant differences among plots (*p* < 0.05, n = 36). The length of the rhombs represents the IQR (distance from the 25th to 75th percentiles). The top horizontal line is Q3 + 1.5*IQR, and the bottom line is Q1–1.5*IQR.

**Figure 2:**
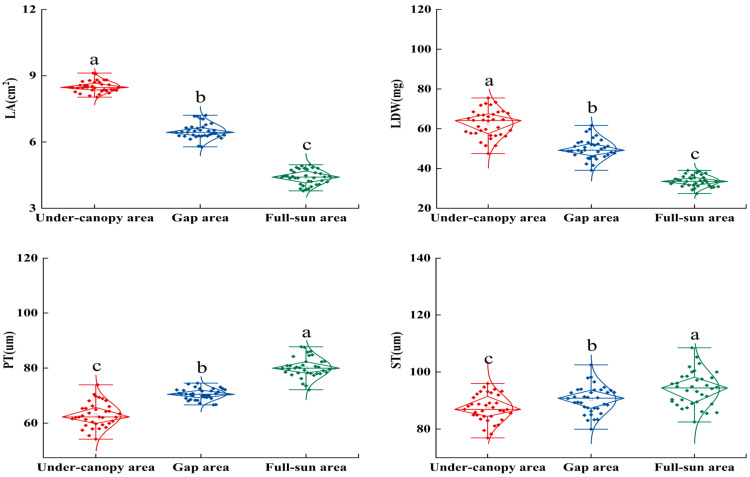
Leaf traits of *Periploca sepium* in response to varying light environments. LA: leaf area; LDW: leaf dry weight; PT: palisade tissue thickness; ST: spongy tissue thickness. Different lowercase letters denote significant differences among plots (*p* < 0.05, n = 36). Additional explanation is required, as detailed below. The length of the rhombs represents the IQR (distance from the 25th to 75th percentiles). The top horizontal line is Q3 + 1.5*IQR, and the bottom line is Q1 – 1.5*IQR.

Considerable variation was observed in the twig and leaf traits of *P. sepium* across different light environments (*p* < 0.05; Figure 1 and Figure 2; Supplementary Tables S1 and S2). The values for TD, TL, TDW, LA, and LDW were consistently highest in the under-canopy area and lowest in the full-sun area, decreasing by 8.23%, 28.28%, 31.03%, 48.24%, and 46.19%, respectively; in contrast, LN, PT, and ST reached their minimum values in the under-canopy area and their maximum in the full-sun area, increasing by 50.94%, 27.26%, and 7.74%, respectively.

### 2.3. Photosynthetic Physiological Traits of Periploca sepium in Response to Varying Light Environments

**Figure 3:**
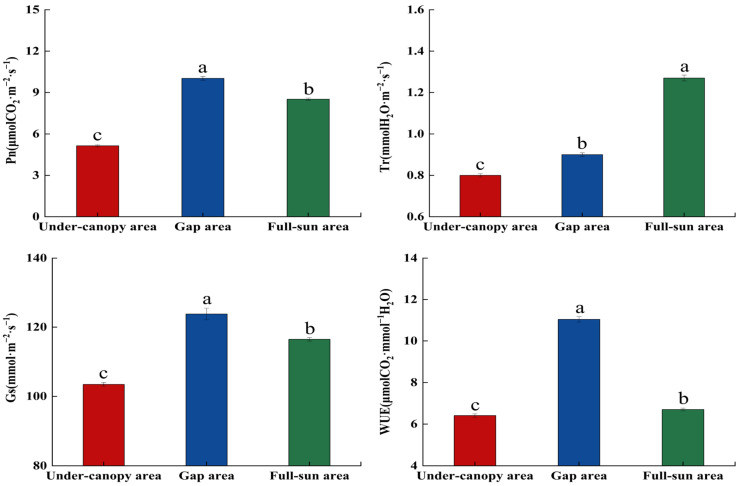
Photosynthetic parameters of *Periploca sepium* in response to varying light environments. Pn: net photosynthetic rate; Tr: transpiration rate; Gs: stomatal conductance; WUE: water use efficiency. Different lowercase letters denote significant differences among plots (*p* < 0.05, n = 36).

**Figure 4:**
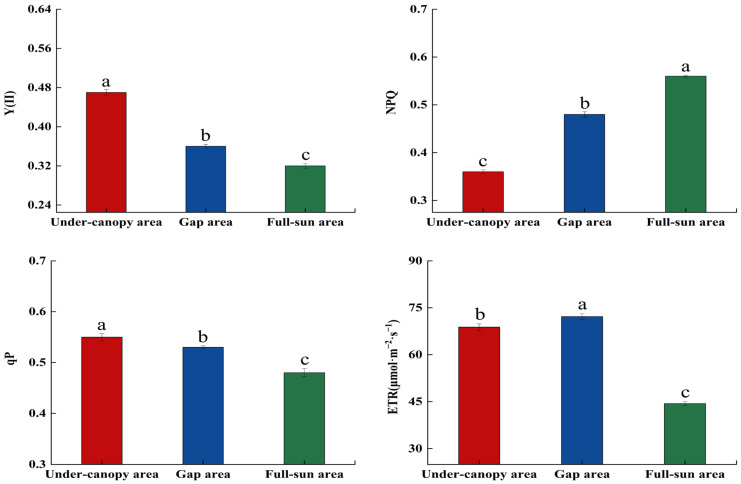
Chlorophyll fluorescence parameters of *Periploca sepium* in response to varying light environments. Y(II): actual photosynthetic efficiency of photosystem II; NPQ: non-photochemical quenching; qP: photochemical quenching; ETR: electron transfer rate of photosystem II. Different lowercase letters denote significant differences among plots. (*p* < 0.05, n = 36).

The photosynthetic parameters and chlorophyll fluorescence parameters of *P. sepium* showed significant changes under different light environments (*p* < 0.05, Figure 3 and Figure 4; Supplementary Tables S3 and S4). The lowest values of Tr and NPQ were recorded in the under-canopy area, while both parameters peaked in the full-sun area. Conversely, the under-canopy area exhibited the highest Y(II) and qP values, while the full-sun area showed the lowest. Pn, Gs, and WUE were maximized in the gap area and minimized in the under-canopy area. As the habitat light conditions changed from the under-canopy area to the full-sun area, Tr and NPQ showed increasing trends, rising by 0.59- and 0.56-fold, while Pn, Gs, WUE, and ETR first increased and then decreased, with overall increases of 65.76%, 12.60%, 4.52%, and 55.10%, respectively. Y(II) and qP decreased by 31.91% and 12.73%, respectively.

### 2.4. Effects of Environmental Factors on Functional Traits of Periploca sepium

**Figure 5:**
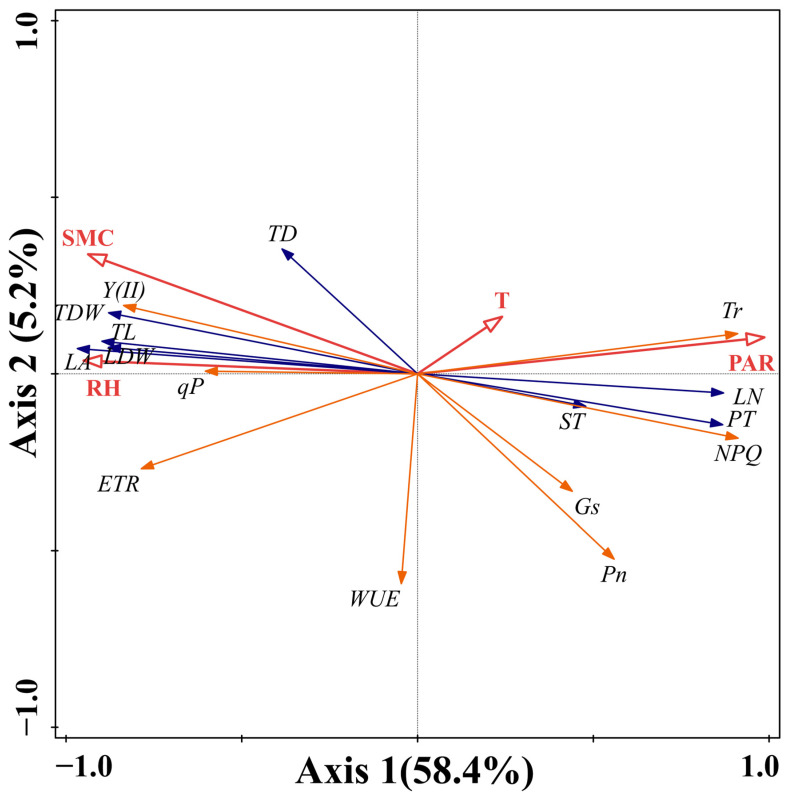
RDA of environmental factors and functional traits of *Periploca sepium*. PAR: photosynthetically active radiation; SMC: soil moisture content; T: air temperature; RH: air relative humidity; TD: twig diameter; TL: twig length; TDW: twig dry weight; LN: leaf number; LA: leaf area; LDW: leaf dry weight; PT: palisade tissue thickness; ST: spongy tissue thickness; Pn: net photosynthetic rate; Tr: transpiration rate; Gs: stomatal conductance; WUE: water use efficiency; Y(II): actual photosynthetic efficiency of photosystem II; NPQ: non-photochemical quenching; qP: photochemical quenching; ETR: electron transfer rate of photosystem II.

The RDA analysis reveals that the first and second axes account for 58.4% and 5.2% of the ecological information, respectively, cumulatively explaining 63.6% of the variation in *P. sepium* traits and highlighting the relationship between *P. sepium*’s functional traits and environmental factors, with Axis 1 being the primary determinant. PAR exhibits the strongest correlation with Axis 1, followed by RH and SMC (Figure 5). PAR gradually increases from left to right along RDA axis 1, while RH and SMC exhibit the opposite trend. The weak correlation between T and Axis 1 implies minimal impact of T on *P. sepium* traits. Monte Carlo tests revealed that PAR and SMC significantly influence *P. sepium* traits (*p* < 0.05).

### 2.5. Relationships Between Twig and Leaf Morphological Traits and Photosynthetic Characteristics of Periploca sepium

**Figure 6:**
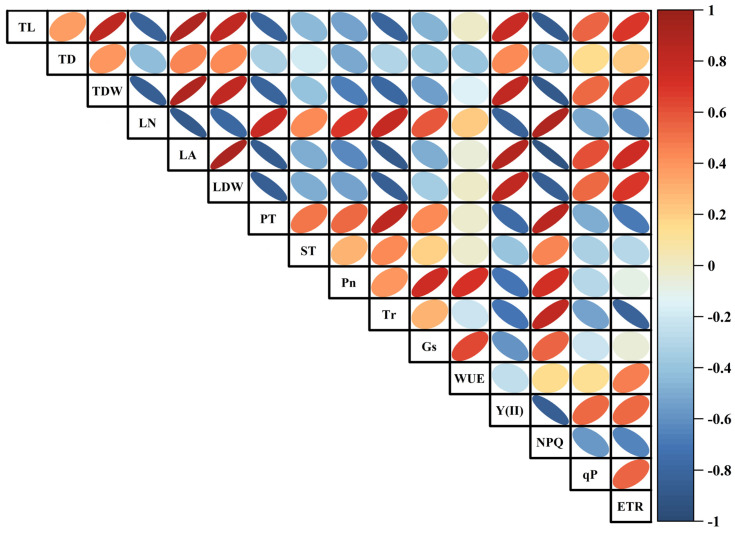
Correlation analysis of twig and leaf morphological traits and photosynthetic characteristics in *Periploca sepium*. TD: twig diameter; TL: twig length; TDW: twig dry weight; LN: leaf number; LA: leaf area; LDW: leaf dry weight; PT: palisade tissue thickness; ST: spongy tissue thickness; Pn: net photosynthetic rate; Tr: transpiration rate; Gs: stomatal conductance; WUE: water use efficiency; Y(II): actual photosynthetic efficiency of photosystem II; NPQ: non-photochemical quenching; qP: photochemical quenching; ETR: electron transfer rate of photosystem II.

The relationships between the twig and leaf morphologies of *P. sepium* and its photosynthetic characteristics were analyzed through Pearson correlation analysis (Figure 6). Results showed that TL was significantly positively correlated with Y(II), qP, and ETR, but significantly negatively correlated with Pn, Tr, Gs, and NPQ (Figure 6; Supplementary Table S5). TD showed a significant positive correlation with Y(II) and a positive correlation with ETR, but a significant negative correlation with Pn, Tr, Gs, WUE, and NPQ. TDW was significantly positively correlated with Y(II), qP, and ETR, yet significantly negatively correlated with Pn, Tr, Gs, and NPQ. In contrast, LN exhibited significant positive correlations with Pn, Tr, Gs, and NPQ, a positive correlation with WUE, and significant negative correlations with Y(II), qP, and ETR. LA and LDW both displayed significant positive correlations with Y(II), qP, and ETR, but negative correlations with Pn, Tr, Gs, and NPQ. PT was significantly positively correlated with Pn, Tr, Gs, and NPQ, yet negatively correlated with Y(II), qP, and ETR. ST demonstrated significant positive correlations with Pn, Tr, and NPQ, a positive correlation with Gs, and negative correlations with Y(II), qP, and ETR.

**Figure 7:**
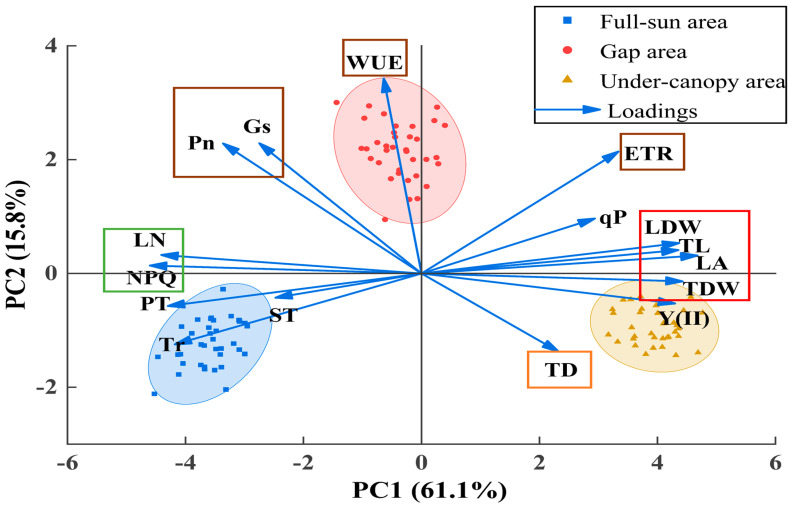
Principal component analysis of twig and leaf morphological traits and photosynthetic characteristics in *Periploca sepium*. TD: twig diameter; TL: twig length; TDW: twig dry weight; LN: leaf number; LA: leaf area; LDW: leaf dry weight; PT: palisade tissue thickness; ST: spongy tissue thickness; Pn: net photosynthetic rate; Tr: transpiration rate; Gs: stomatal conductance; WUE: water use efficiency; Y(II): actual photosynthetic efficiency of photosystem II; NPQ: non-photochemical quenching; qP: photochemical quenching; ETR: electron transfer rate of photosystem II. Traits within the red box show high positive loadings on the first principal component, whereas those in the green box exhibit high negative loadings. Traits within the brown box show high positive loadings on the second principal component, while those in the orange box exhibit high negative loadings.

The principal component analysis of twig and leaf morphological traits and photosynthetic physiological characteristics of *P. sepium* under heterogeneous light environments showed that PC1 and PC2 explained 61.1% and 15.8% of the variance, respectively, for a cumulative total of 76.9% (Figure 7). The first principal component was associated with high positive loadings of LA, TDW, LDW, and TL, and high negative loadings of NPQ and LN. The second principal component was associated with high positive loadings of WUE, Gs, Pn, and ETR, and high negative loadings of TD.

## 3. Discussion

Twig and leaf traits form the fundamental basis for balancing photosynthetic efficiency and resource allocation strategies, reflecting the coordinated combination of structure and function that plants develop to optimize photosynthetic performance in heterogeneous habitats [15,38]. In this study, we found that, from the under-canopy area to the full-sun area, both twig and leaf morphological traits and photosynthetic physiological characteristics of *P. sepium* changed significantly and were strongly correlated with each other. The results of this study show that the adaptation of *P. sepium* to heterogeneous light environments follows the expected pattern of coordinated variation in twig and leaf morphologies and photosynthetic physiology.

### 3.1. Responses of Twig and Leaf Morphologies and Photosynthetic Characteristics of Periploca sepium to the Light Environment in the Under-Canopy Area

Twig and leaf morphologies and arrangement patterns can form covariant relationships with heterogeneous habitats, enabling plants to adapt to their environments most economically [17,18]. In the under-canopy area, the canopy of *Populus simonii* blocks most direct sunlight, making light intensity the main limiting factor for the growth of *P. sepium*. The height and crown width of the *P. sepium* population reach their maxima in this habitat (Table 1). To adapt to low-light environments, *P. sepium* develops a configuration in which a few large leaves are evenly distributed along long and thick twigs (Figure 2 and Figure 3); actual photosynthetic efficiency of photosystem II and photochemical quenching are highest, while non-photochemical quenching is lowest (Figure 5). Twig and leaf morphological traits are significantly correlated with photosynthetic physiological parameters (Figure 6 and Figure 7), a result which can be attributed to the following factors: (1) Larger leaves increase the light-capturing surface area and produce more organic matter with which to sustain growth and development [19]; meanwhile, relatively thin palisade and spongy tissues enhance the efficiency of light-quantum capture [39]. Reducing leaf number offsets the increased cost of constructing larger leaves, while even distribution along the twig minimizes internal shading [40,41]. (2) Increased twig length expands the spatial distribution of leaves, whereas greater twig diameter not only provides mechanical support for large leaves but also enhances water-transport capacity through higher vessel density and diameter, thus ensuring efficient physiological function [42]. (3) Shading reduces both leaf surface and soil temperatures, decreasing stomatal conductance and transpiration rate in *P. sepium* leaves, and insufficient light capture results in the lowest net photosynthetic rate in this habitat; in the limited light conditions of the under-canopy area, leaves allocate more absorbed light energy to photochemical reactions, with actual photosynthetic efficiency of photosystem II and photochemical quenching reaching their highest levels. Therefore, *P. sepium* optimizes its twig and leaf configurations to enhance light capture efficiency, maintaining carbon assimilation capacity and reproductive fitness under low-light conditions.

### 3.2. Responses of Twig and Leaf Morphologies and Photosynthetic Characteristics of Periploca sepium to the Light Environment in the Full-Sun Area

Twig and leaf traits serve as crucial links in coordinating light energy acquisition and material conversion in photosynthetic physiology, reflecting the dynamic equilibrium strategy of structure and metabolism that plants adopt to maximize photosynthetic efficiency under variable environmental stresses [43]. In the full-sun area, increased sunlight accelerates soil moisture evaporation, making water the key limiting factor for *P. sepium* growth. The height and crown width of the *P. sepium* population attain their minimum values in this habitat (Table 1), where *P. sepium* evolved numerous small leaves clustered on short and thick twigs (Figure 2 and Figure 3). The actual photosynthetic efficiency of photosystem II and photochemical quenching are lowest, while non-photochemical quenching is highest (Figure 5). Significant correlations exist between the twig and leaf morphological traits of *P. sepium* and its photosynthetic physiological parameters (Figure 6 and Figure 7), which can be attributed to the following factors: (1) Small leaves reduce the costs of plant respiration and transpiration, capturing light energy while minimizing water loss and preventing decreases in cellular water potential and turgor pressure [44,45]; small leaves have a short unfolding period and a higher heat exchange coefficient, with low thermal and material exchange resistance at the leaf margins, granting them a competitive advantage under high-light conditions [19,46]. Greater thicknesses of palisade and spongy tissue improve both leaf water retention and photosynthetic efficiency (Figure 2) [47]. (2) *P. sepium* shortens its twigs to cut the transport distances for water and nutrients from root to leaf, while the greater twig diameter secures the water supply to numerous small leaves and stabilizes their physiological performance [48]. (3) Under high-light conditions, *P. sepium* reduces its stomatal conductance to curb transpiration-related water loss, yet this also restricts carbon dioxide influx and drives net photosynthetic rate downward; *P. sepium* prioritizes allocating photosynthetic products to protect photosynthetic organs against intense light stress, resulting in relatively low actual photosynthetic efficiency of photosystem II and photochemical quenching values, as well as maximized non-photochemical quenching. Therefore, in water-limited full-sun area, *P. sepium* reduces respiratory and transpiration costs through covariation in twig and leaf traits, enhancing water retention and photosynthetic efficiency to successfully adapt to habitat stress.

### 3.3. Responses of Twig and Leaf Morphologies and Photosynthetic Characteristics of Periploca sepium to the Light Environment in the Gap Area

The plasticity of twig and leaf traits serves as a key strategy for plants to optimize resource acquisition and mitigate environmental stress in heterogeneous habitats [49,50]. As a transitional zone, the gap area exhibits a 29.36% increase in photosynthetically active radiation relative to the under-canopy area, while soil moisture content decreases by 24.25%. The height and crown width of the *P. sepium* population fall between those of the under-canopy area and full-sun area (Table 1). In this habitat, *P. sepium* develops long and thick twigs bearing dense small leaves, and significant correlations exist between twig–leaf morphologies and photosynthetic physiological parameters (Figure 6 and Figure 7), a result which can be attributed to the following factors: (1) With the markedly improved light environment of the gap area, *P. sepium* only needs to develop small leaves to meet its light requirements for growth; these small leaves also reduce water deficit stress caused by excessive transpiration. Additionally, long twigs enhance the plant’s ability to capture vertical light resources, while thick twigs ensure efficient water transport to support normal transpiration [51]. In this habitat, the leaf and twig morphologies of *P. sepium* are intermediate, between those of the under-canopy and full-sun areas, likely reflecting a trade-off in resource allocation and consistent with the functional balance hypothesis [52]. (2) The suitable light in the gap area promotes stomatal opening, and the maximum stomatal conductance accelerates the supply of carbon dioxide inside the leaves, which, in turn, maximizes net photosynthetic rate; simultaneously, the synchronous thickening of palisade and spongy tissues extends the water vapor diffusion pathway within the leaf, reducing transpiration rate and maximizing the leaf’s water use efficiency. (3) The gap area has moderate thermal and hydrological conditions, resulting in relatively low survival stress for *P. sepium*. In this habitat, *P. sepium* protects its photosynthetic organs from photoinhibition damage by enhancing leaf heat dissipation, while the high electron transfer rate of photosystem II ensures efficient photochemical reactions. Therefore, through the synergistic plasticity of twig and leaf morphologies and photosynthetic physiology, *P. sepium* in the gap area achieves a carbon investment trade-off between plant growth and defense.

## 4. Materials and Methods

### 4.1. Study Site

**Figure 8:**
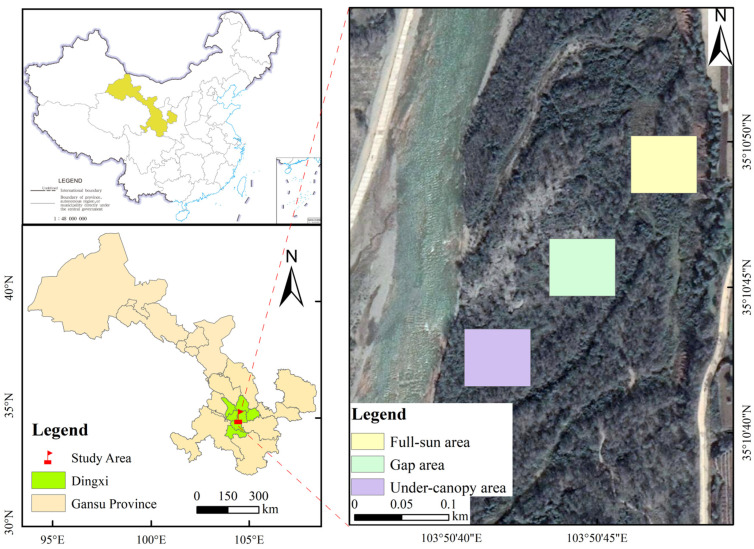
Location of study area and field sampling plots.

The field experiments were carried out within Taohe National Wetland Park in Lintao County, Gansu Province (103°45′43″–103°50′55″ E, 35°05′27″–35°15′58″ N), an area situated at the junction of the Qinghai–Tibet and Loess Plateaus, with altitudes between 1880 and 1930 m (Figure 8); this region belongs to the mid-temperate semi-arid and semi-humid convergence climate zone. Mean annual temperature and precipitation are recorded as 7.7 °C and 495.1 mm, respectively. The annual sunshine real number 2501.8 h. Soil types are mainly red loam, tidal soil, and sandy soil [14].

### 4.2. Field Experiment Methods and Designs

The field experiment was conducted from 15 to 30 August 2023. After several field investigations in Taohe National Wetland Park, the native Taohe riparian forest plant *P. sepium* was selected for this study. Field investigations and data analyses indicate that interactions between tree and shrub communities in the Taohe riparian forest reduce understory shrubs’ light resources, altering the *P. sepium* community environment and resulting in microhabitat-specific biological characteristics of the *P. sepium* population. Based on the shade conditions of the *Populus simonii* canopy and the distance from trees, the *P. sepium* community was classified into the following three different habitats: (1) the under-canopy area, defined as locations beneath a completely closed or intersecting canopy; (2) the gap area, referring to forest clearings with diameter of at least 5 m or width equal to the tree height, which receive direct sunlight; and (3) the full-sun area, consisting of open spaces located more than one tree height from the forest edge. Six 5 m × 5 m quadrats were established per habitat, resulting in a total of 18 quadrats. Environmental characteristics, biological characteristics, twig and leaf morphological traits, and photosynthetic physiological characteristics were measured in each habitat.

#### 4.2.1. Determination of Gas Exchange Parameters

Well-growing current-year twigs were randomly selected from the middle and upper parts of the canopy in four directions for each sampled plant, and three fully expanded leaves were selected from the fifth leaf from the top of the labeled twig. Photosynthetic parameters, including net photosynthetic rate (Pn), transpiration rate (Tr), stomatal conductance (Gs), and water use efficiency (WUE), were measured using a GFS-3000 portable photosynthetic measurement system (Heinz Walz GmbH, Effeltrich, Germany) in the morning (8:00–12:00). During the measurement, an artificial red–blue light source was used. The measurement conditions were set as follows: flow rate of 750 ± 10 μmmol·s^−1^, CO_2_ concentration of 430 ± 10 μmmol·mol^−1^, temperature of 15 ± 5 °C, relative humidity of 60–70%, and photosynthetic photon flux density (PPFD) of 1200 μmol·m^−2^·s^−1^ [53].

#### 4.2.2. Determination of Chlorophyll Fluorescence Parameters

Chlorophyll fluorescence parameters were measured using the IMAGING-PAM chlorophyll fluorometer system (Heinz Walz GmbH, Effeltrich, Germany); these parameters included the quantum efficiency of PSII photochemistry [Y(II)], photochemical quenching coefficient (qP), non-photochemical quenching coefficient (NPQ), and electron transport rate (ETR). All measurements were taken after a 30 min dark adaptation period [54,55].

#### 4.2.3. Determination of Twig and Leaf Morphological Characteristics

After the photosynthetic physiological measurements were completed, twig samples were collected, immediately placed in sealed plastic bags, and transported to the laboratory for further analysis. Leaf number (LN) per twig was quantified via direct counting, and leaf area (LA) was measured with a CI-202 portable leaf area meter (CID Bio-Science, Camas, WA, USA). Twig length (TL) and diameter (TD) were determined using a metric ruler (1 mm accuracy) and a vernier caliper (0.02 mm accuracy), respectively. Three mature leaves from the middle part of each twig were selected, and 1 cm × 1 cm square leaf pieces were cut from both sides of the midrib. The pieces were then placed into FAA fixative and quickly brought back to the laboratory for the determination of leaf anatomical structure. The collected samples were sectioned using the paraffin sectioning method and stained with safranin and fast green [56]. The sections were observed under the light microscope (Olympus BX53, Shinjuku, Tokyo, Japan), and the thicknesses of the palisade tissue (PT) and spongy tissue (ST) were measured using ImageJ software (version 1.8.0). Finally, twigs and leaves were oven-dried at 80 °C to constant mass, and then weighed separately using an electronic balance.

#### 4.2.4. Measurement of Environmental Factors

During the measurement of photosynthetic parameters, a handheld photometer (3415F, Spectrum Technologies, Aurora, IL, USA) was employed to measure the photosynthetically active radiation (PAR) of each plot. The handheld weather station (NK4000, Kestrel Meters, Boothwyn, PA, USA) was used to measure the air humidity (RH) and air temperature (T) of each sample. At each sampling point, soil samples from 0–20 cm, 20–40 cm, and 40–60 cm depths were collected, cleaned of rocks and roots, mixed evenly by layer, and weighed. Soil moisture content (SMC) was determined by drying the samples at 105 °C until constant weight was achieved.

### 4.3. Data Analysis

One-way ANOVA was performed to compare environmental characteristics, biological characteristics, twig and leaf morphological traits, and photosynthetic physiological characteristics among different habitats (α = 0.05). Subsequently, redundancy analysis (RDA) was used to examine how environmental factors influence plant functional traits. Pearson correlation analysis and principal component analysis were conducted to examine the relationships between twig and leaf morphological traits of *P. sepium* and its photosynthetic characteristics. All analyses were carried out using SPSS 22.0 and Canoco 5.0, with plots generated using Origin 2021 and Canoco 5.0.

## 5. Conclusions

Under different habitat conditions, *P. sepium* exhibits distinct adaptive differences in twig and leaf morphological traits, as well as photosynthetic physiological characteristics. In the under-canopy area, the plants develop long and thick twigs with sparsely distributed large leaves; meanwhile, they demonstrate coordinated changes in photosynthetic physiology, characterized by higher actual photosynthetic efficiency of photosystem II and lower non-photochemical quenching, forming an effective adaptation mechanism to the weak-light environment. Conversely, in the full-sun area, the plants adopt the opposite synergistic regulation strategy to adapt to the high-light environment, reflecting the survival strategy of riparian forest plants, which have adapted over long periods to heterogeneous light environments. Heterogeneous habitats alter the quantities of resources available to plants. Insufficient resource supply causes changes in plant twig and leaf morphologies, which, in turn, affect photosynthetic physiological processes. The synergistic responses between twig and leaf morphologies and photosynthetic traits reflect plants’ ecological adaptation strategies to acquire heterogeneous resources efficiently. The result of this study reveals the above-ground strategies of *P. sepium* for resource capture and utilization under heterogeneous light environments. Plant acclimation depends on the coordination and trade-offs between above- and below-ground traits. Therefore, subsequent research will be focused on quantifying the below-ground functional traits of *P. sepium* to provide a comprehensive analysis of its adaptive strategies in heterogeneous light environments.

## Data Availability

The data used in the present work have been listed in the Supplementary Materials.

## Supplementary Materials

The following supporting information can be downloaded at: https://www.mdpi.com/article/10.3390/plants15020179/s1, Table S1: Twig traits of *Periploca sepium* in response to varying light environments; Table S2: Leaf traits of *Periploca sepium* in response to varying light environments; Table S3: Photosynthetic parameters of *Periploca sepium* in response to varying light environments; Table S4: Chlorophyll fluorescence parameters of *Periploca sepium* in response to varying light environments; Table S5: Correlation analysis of twig and leaf morphological traits and photosynthetic characteristics in *Periploca sepium*.
