# Supplementary material for: Twig and Leaf Morphological Traits and Photosynthetic Physiological Characteristics of Periploca sepium in Response to Different Light Environments in Taohe Riparian Forests
Source: Plants (Basel). 2026 Jan 7;15(2):179. doi: 10.3390/plants15020179 (PMC12844625; doi:10.3390/plants15020179)
Supplement: Supplementary file 1 [file plants-15-00179-s001.zip › plants-4062477-supplementary.pdf]

Table S1 Twig traits of *Periploca sepium* under varying light environments (mean ± *SE*)

Plot	TD (mm)	TL (cm)	TDW (g)	LN (No.)
Under canopy area	1.58 ± 0.019a	20.05 ± 0.232a	0.58 ± 0.007a	18.00 ± 0.214c
Gap area	1.43 ± 0.014b	17.44 ±0.151b	0.47 ±0.003b	24.00 ± 0.320b
Full sun area	1.45 ± 0.018b	14.38 ±0.163c	0.40 ± 0.006c	27.17 ± 0.300a

Different lowercase letters in the same column indicate significant differences among plots ($P < 0.05$). TD: twig diameter; TL: twig length; TDW: twig dry weight; LN: leaf number.

Table S2 Leaf traits of *Periploca sepium* under varying light environments (mean ± *SE*)

Plot	LA (cm^2)	LDW (mg)	PT (mm)	ST (mm)
Under canopy area	8.50 ± 0.042a	62.55 ±1.144a	63.03 ± 0.740a	87.26 ± 0.791c
Gap area	6.49 ± 0.057b	49.81 ±0.824b	70.48 ±0.328b	90.30 ± 0.803b
Full sun area	4.40 ± 0.055c	33.66 ±1.244c	80.21 ± 0.601c	94.01 ± 1.003a

Different lowercase letters in the same column indicate significant differences among plots ($P < 0.05$).LA: leaf area; LDW: leaf dry weight; PT: palisade tissue thickness; ST: spongy tissue thickness.

Table S3 Photosynthetic parameters of *Periploca sepium* under varying light environments (mean ± *SE*)

Plot	Pn ($\mu molCO_2 \cdot m^{-2} \cdot s^{-1}$)	Tr($mmolH_2O \cdot m^{-2} \cdot s^{-1}$)	Gs($mmol \cdot m^{-2} \cdot s^{-1}$)	WUE($\mu molCO_2 \cdot mmol^{-1} \cdot H_2O$)
Under canopy area	5.14± 0.068c	0.80 ± 0.008c	103.45 ± 0.487c	6.41 ± 0.082c
Gap area	10.03 ±0.140a	0.90 ± 0.009b	123.80 ± 1.688a	11.04 ± 0.127a
Full sun area	8.52 ± 0.084b	1.27 ± 0.014a	116.48 ± 0.500b	6.70 ± 0.065b

Different lowercase letters in the same column indicate significant differences among plots ($P < 0.05$). Pn: net photosynthetic rate; Tr: transpiration rate; Gs: stomatal conductance; WUE: water use efficiency.

Table S4 Chlorophyll fluorescence parameters of *Periploca sepium* under varying light environments (mean ± *SE*)

Plot	Y(II)	NPQ	qP	ETR($\mu mol \cdot m^{-2} \cdot s^{-1}$)
Under canopy area	0.47± 0.006a	0.36 ± 0.004c	0.55 ± 0.007a	68.82 ± 1.032b
Gap area	0.36 ±0.004b	0.48 ± 0.006b	0.53 ± 0.003b	72.16 ± 0.940a

Full sun area	0.32 ± 0.005c	0.56 ± 0.002a	0.48 ± 0.008c	44.37 ± 0.620c

Different lowercase letters in the same column indicate significant differences among plots ($P < 0.05$). Y(II): actual photosynthetic efficiency of photosystem II; NPQ: non-photochemical quenching; qP: photochemical quenching; ETR: electron transfer rate of photosystem II.

Table S5 Correlation analysis between twig and leaf morphological traits and photosynthetic characteristics of *Periploca sepium*

	TL	TD	TDW	LN	LA	LDW	PT	ST	Pn	Tr	Gs	WUE	Y(II)	NPQ	qP	ETR
TL	1															
TD	0.37**	1														
TDW	0.83**	0.39**	1													
LN	-0.82**	-0.43**	-0.85**	1												
LA	0.89**	0.44**	0.90**	-0.89**	1											
LDW	0.81**	0.43**	0.81**	-0.80**	0.90**	1										
PT	-0.82**	-0.33**	-0.82**	0.77**	-0.87**	-0.85**	1									
ST	-0.46**	-0.20*	-0.41**	0.43**	-0.48**	-0.50**	0.50**	1								
Pn	-0.53**	-0.51**	-0.68**	0.69**	-0.63**	-0.53**	0.53**	0.29**	1							
Tr	-0.81**	-0.31**	-0.78**	0.78**	-0.88**	-0.85**	0.83**	0.44**	0.40**	1						
Gs	-0.47**	-0.39**	-0.54**	0.69**	-0.49**	-0.36**	0.42**	0.19*	0.75**	0.29**	1					
WUE		-0.40**	-0.16	0.20*	-0.05	-0.003	-0.04	-0.04	0.70**	-0.22*	0.63**	1				
Y(II)	0.77**	0.42**	0.81**	-0.83**	0.86**	0.80**	-0.77**	-0.39**	-0.71**	-0.71**	-0.60**	-0.26**	1			
NPQ	-0.87**	-0.45**	-0.89**	0.88**	-0.93**	-0.85**	0.85**	0.45**	0.72**	0.81**	0.55**	0.16	-0.85**	1		
qP	0.56**	0.15	0.52**	-0.51**	0.60**	0.53**	-0.49**	-0.33**	-0.29**	-0.53**	-0.21*	0.13	0.52**	-0.57**	1	
ETR	0.68**	0.21*	0.61**	-0.58**	0.74**	0.68**	-0.69**	-0.29**	-0.10	-0.82**	-0.06	0.47**	0.52**	-0.64**	0.55**	1

TD: twig diameter; TL: twig length; TDW: twig dry weight; LN: leaf number; LA: leaf area; LDW: leaf dry weight; PT: palisade tissue thickness; ST: spongy tissue thickness; Pn: net photosynthetic rate; Tr: transpiration rate; Gs: stomatal conductance; WUE: water use efficiency; Y(II): actual photosynthetic efficiency of photosystem II; NPQ: non-photochemical quenching; qP: photochemical quenching; ETR: electron transfer rate of photosystem II. *$P < 0.05$ (bilateral); ** $P < 0.01$ (bilateral).
